# Clinical Features, Diagnosis, and Treatment Strategies of Gastrointestinal Diaphragm Disease Associated with Nonsteroidal Anti-Inflammatory Drugs

**DOI:** 10.1155/2016/3679741

**Published:** 2016-03-28

**Authors:** Yan-Zhi Wang, Gang Sun, Feng-Chun Cai, Yun-Sheng Yang

**Affiliations:** Department of Gastroenterology and Hepatology, Chinese People's Liberation Army General Hospital, Beijing 100853, China

## Abstract

*Background*. To demonstrate the clinical features, diagnosis, and treatment of nonsteroidal anti-inflammatory drug- (NSAID-) induced diaphragm disease (DD).* Methods*. A literature search between January 1973 and August 2015 was undertaken. The clinical data of patients with NSAID-induced DD were recorded and analyzed.* Results*. 159 patients were included. The ratio of male to female was 1 : 2.3; the mean age was 65 ± 11 years. The most common clinical manifestations were gastrointestinal bleeding and obstruction. 121 (84%) patients took traditional NSAIDs. The durations of NSAIDs use ranged from 2 to 300 months. A majority (59.7%) of DD were seen in the small bowel, were seen secondly in the colon (30.2%), and were mainly located in the ileum (57.9%) and right colon (91.7%), respectively. 80% of patients had multiple diaphragms. 41.5% of small bowel DD were diagnosed preoperatively by capsule endoscopy and/or double-balloon enteroscopy, 52.1% at laparotomy. Nearly 75% of patients underwent surgery, endoscopic balloon dilation was performed in 22 patients, and NSAIDs were withdrawn in 53 patients.* Conclusions*. NSAID-induced DD is relatively rare. The small bowel is most commonly involved. Preoperative diagnosis of small bowel DD is relatively difficult. Discontinuation of the NSAIDs is recommended, surgical resection is the main treatment presently, and endoscopic balloon dilation should be considered as an alternative therapy.

## 1. Introduction

Since the synthesis of aspirin in 1899, nonsteroidal anti-inflammatory drugs (NSAIDs) have been one of the most widely prescribed drugs in the world for defervescence, analgesia, and the therapy of inflammatory conditions including osteoarthritis and rheumatoid arthritis [1]. New, promising fields of application in cancer prophylaxis have also arisen [2]. Moreover, their widespread and sometimes uncontrolled use is promoted by their over-the-counter availability in many countries. It is indicated by a 2010 National Health Interview Survey (NHIS) that around 43 million adults (19.0%) in the United States took aspirin at least three times per week for more than 3 months (i.e., regular users), and more than 29 million adults (12.1%) were regular users of NSAIDs. Compared with 2005, this was an overall increase of 57% in aspirin use and 41% in NSAID use [3].

It has been well known that NSAIDs could cause gastrointestinal (GI) inflammation, ulceration, bleeding, and perforation [4]. But it has not been widely recognized that NSAIDs also can cause other types of lesions, for example, formation of diaphragm-like stricture [5, 6]. Cases of small bowel strictures associated with NSAIDs have been reported since the 1970s [7]. Recurrent small bowel obstruction associated with piroxicam was again reported in 1987 by Sukumar who mentioned diaphragm-like strictures as the cause of the obstruction [8]. However, the “diaphragm disease (DD)” was first termed by Lang et al. in 1988 [9]. In their report, 7 cases of DD were identified, the clinicopathological features and its strong relationship to NSAID were described, and the possible mechanisms were discussed. The first report of NSAID-induced colonic DD was a letter by Sheers and Williams in 1989 [10].

The relationship between NSAIDs use and GI inflammation and ulceration has been well demonstrated by large studies. However, the current papers about the diaphragm-like stricture induced by NSAIDs are mostly case reports. There are several reviews on NSAID-induced DD, but only involving the small intestine or colon [11, 12]. In this paper we describe the clinical features, diagnosis, and treatment strategies of NSAID-induced DD involving stomach, duodenum, small bowel, and colon and hope to further the clinical awareness of this entity which may become increasingly important in the era of widespread use of NSAIDs.

## 2. Methods

### 2.1. Literature Search and Management Procedure

**Figure 1:**
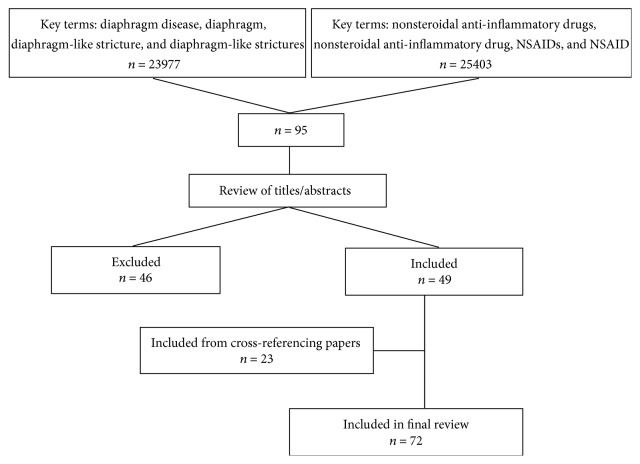
Literature search and management procedure.

A literature search was undertaken using the terms “diaphragm disease” or “diaphragm” or “diaphragm-like stricture” or “diaphragm-like strictures” in combination with “nonsteroidal anti-inflammatory drugs” or “nonsteroidal anti-inflammatory drug” or “NSAIDs” or “NSAID”. PubMed was consulted to search for papers published between January 1973 and August 2015. Titles were reviewed and any papers with nonrelevant titles were excluded. Abstracts of the remaining papers were subsequently systematically reviewed. If there was no abstract, the paper would have been browsed. The paper would have been excluded if the abstract was not related to DD or DD unrelated to NSAIDs or not written in English. All published papers (including articles, reviews, case reports, and letters) referring to NSAIDs-induced DD were included. The references of each paper were consulted and any relevant papers were also reviewed for inclusion. Finally, the remaining 72 papers were included in the present paper. The literature search and management procedure is presented in Figure 1.

### 2.2. Data Extraction

Clinical data of patients extracted included age at onset; gender; clinical presentation; type, dosage, and duration of NSAIDs use; examination; diagnostic method; location and number of diaphragm-like strictures; management.

### 2.3. Statistical Analysis

Descriptive statistics for continuous variables (age, duration of NSAIDs use) and discrete variables (number of diaphragm-like strictures) were presented as mean values ± SD, and minimum and maximum values and categorical variables were presented as percent.

## 3. Results

### 3.1. Age, Gender, and Clinical Manifestations

159 patients (including our one case) with NSAIDs-induced DD were analyzed. 106 patients were female and only 47 were male; with gender unknown in six cases, the ratio of male to female is 1 : 2.3. The mean age was 65 ± 11 years (age range, 37–90 years).

The most common clinical manifestations were GI bleeding and obstruction. 102 (65.8%) and 113 (72.9%) patients presented with GI bleeding and obstruction, respectively. The other clinical manifestations were shown in Table 1.

### 3.2. Type and Duration of NSAIDs Use

57 patients took various NSAIDs, 87 patients took one kind of NSAID, and the NSAIDs taken in the remaining 15 patients were unspecified. 121 (84%) patients took traditional NSAIDs, including diclofenac used most commonly in 47 cases (slow release agent in 16 cases, suppository in 2 cases). 38 (26.4%) patients took selective cyclooxygenase-2 (COX-2) inhibitors. Details of the other NSAIDs were shown in Table 2. Dramatically, more than 40 tablets of compound aminopyrine phenacetin were taken by mistake while drunk one month prior to the onset in our case.

Of the 138 patients in whom durations of NSAID use were reported, 133 patients had been taking NSAIDs for more than one year. Of the 92 patients in whom durations of NSAID use were specified, the varying durations ranged from 2 to 300 months and the mean duration was 79 ± 71 months.

### 3.3. Location and Number of Diaphragm-Like Strictures

**Figure 2:**
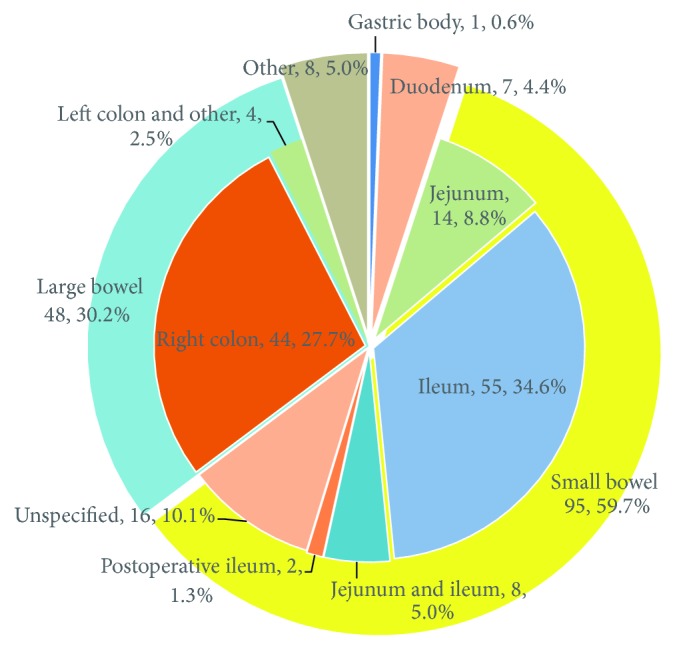
Location of diaphragm-like stricture. Postoperative ileum proximal to the ileal-sigmoid anastomosis (one case) and bypassed ileal segment (one case). Left colon and other included descending colon (one case), lower sigmoid (one case), rectosigmoid junction (one case), and ileocaecal valve, ascending colon, transverse colon, and descending colon were all involved in one case. Other locations included jejunum and duodenum (3 cases); jejunum, duodenum, and pylorus (one case); terminal ileum, ileocaecal valve, caecum, and ascending colon (one case); terminal ileum and ascending colon (one case); terminal ileum and ileocaecal junction (one case); terminal ileum and ileocaecal valve (one case).

The diaphragms were randomly distributed throughout the whole GI tract (Figure 2), but majority (95, 59.7%) were seen in the small bowel, 48 (30.2%) cases in the colon. Small bowel DD was mainly located in the ileum (55, 57.9%). Colonic DD were seen anywhere along the colon from caecum to rectosigmoid junction, but a majority (44, 91.7%) occurred in the right colon and was mainly located in the ascending colon. Of the 123 patients in whom the number of diaphragms was reported, multiple diaphragms were detected in 98 (80%) patients.

### 3.4. Diagnosis and Treatment

**Figure 3:**
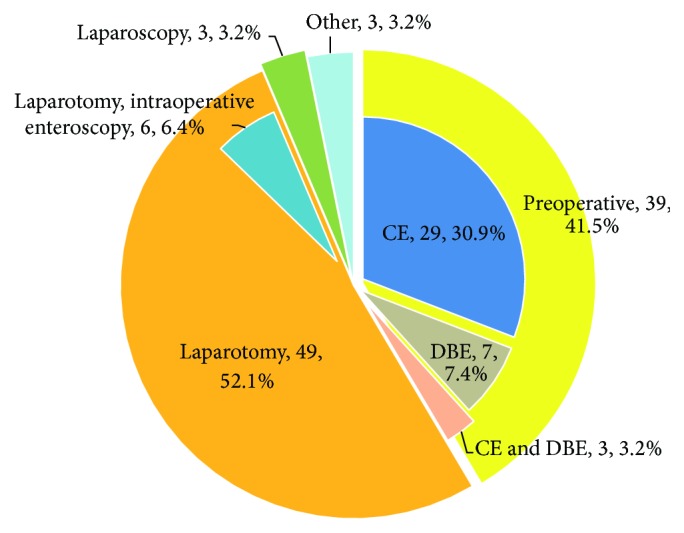
Diagnostic methods. Other methods included autopsy (one case), small bowel enema (one case), and sigmoidoscopy (one case).

Examination methods include endoscopy, gastrointestinal radiology, and laparotomy (Table 3). Gastric and duodenal DD in 7 patients were diagnosed by esophagogastroduodenoscopy (EGD); only one case had DD in duodenum afferent limb diagnosed by double-balloon enteroscopy (DBE). The majority (92%) of colonic DD were diagnosed by colonoscopy. With regard to small bowel DD (Figure 3), preoperative diagnosis was made by CE and/or DBE in 39 (41.5%) patients. In 49 (52.1%) patients, DD was diagnosed by laparotomy. In all 36 patients who underwent CE, retained capsule was retrieved by laparotomy in 31 (86%) patients and by DBE in 4 patients; the capsule was excreted spontaneously in one patient.

**Figure 4:**
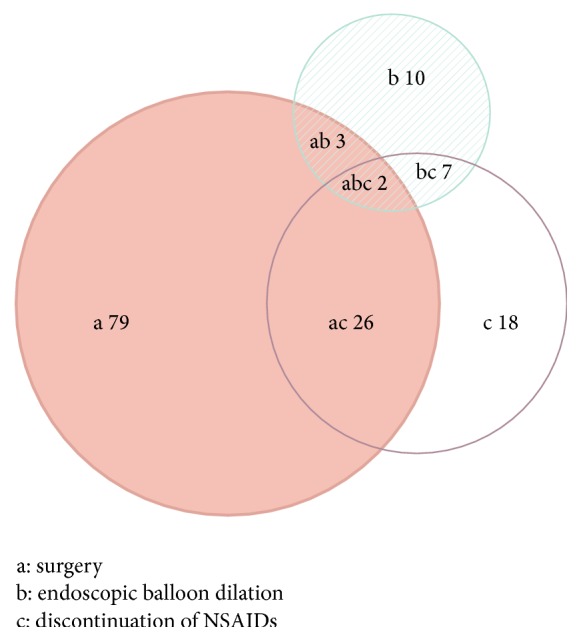
Treatment for patients with NSAIDs-induced DD.

Of the 150 patients with the treatment methods available (Figure 4), nearly 75% of patients underwent surgery. Therapeutic endoscopy was performed in 24 cases, including endoscopic balloon dilation in 22 cases (with placement of a metal stent in our case), incise using a standard sphincterotome in 2 cases. NSAIDs were withdrawn in 53 patients, discontinuation of NSAIDs was the only treatment in 18 patients, and the other 35 patients discontinued NSAIDs as part of the treatment regimen. 38 patients underwent combined therapy.

## 4. Discussion

Gastrointestinal diaphragm-like stricture, also called diaphragm disease, is a relatively rare NSAID-induced complication. It is reported that in 2% of patients taking conventional NSAIDs on a long-term basis, small bowel DD developed [13]. But with the ageing of society, the widespread use of NSAIDs such as aspirin in ischaemic heart disease and arthritis, and increasingly recent recognition, the incidence of DD is on the rise and seems likely to increase in the future; the current actual incidence of DD is still unknown.

DD is probably more common in middle-aged and elderly patients, as they are the most likely to take NSAIDs. The mean age at presentation is 65 ± 11 years in our study. The disease has an obvious female preponderance with ratio of 3 : 1 (2.3 : 1 in our study) presumably due to their higher incidence of chronic diseases requiring long-term analgesic and anti-inflammatory therapy, such as rheumatoid arthritis and osteoarthritis [14]. Clinical manifestations of the DD are nonspecific and insidious, including abdominal pain, vomiting and other obstructive symptoms, loss of blood and protein (overt GI bleeding, anemia, positive fecal occult blood, hypoalbuminemia, and protein-losing enteropathy), diarrhea, constipation, changes in bowel habits, and weight loss [15–17]. Our study demonstrates that the most frequent clinical presentations are GI bleeding and obstructive symptoms as seen in our study (4 cases) and it rarely presented as acute abdomen due to obstruction and/or subsequent perforation [18].

The exact pathogenesis of NSAID-induced DD remains obscure. However, the mechanisms of gastrointestinal damage (such as ulceration) caused by NSAID have been studied and discussed extensively [19–23]. It has been suggested that mucosal damage, for example, circumferential ulceration, could be the precursor of DD [24, 25]. The subsequent reparative process would cause submucosal inflammation and fibrosis. In the healing phase, submucosal granulation tissue matures into collagenous scar tissue; then these rings of scar tissue contract, like drawstrings across the bowel lumen, eventually form diaphragm-like strictures. Moreover, it is paradoxical that despite the wide use of NSAIDs and the high prevalence of NSAID-induced GI inflammation, these lesions can then progress to diaphragm-like strictures only in a few patients. The exact determinants of susceptibility remain unknown. Recently, it has been found that CYP2C9*∗*3 SNPs were significantly associated with an increased risk for DD [26].

The relative risks of the different NSAIDs are not very clear and many studies have shown that selective COX-2 inhibitors may be significantly less injurious to gastrointestinal tract than traditional NSAIDs [26]. As shown in Table 2, 121 (84%) patients took traditional NSAIDs, and 38 (26.4%) patients took selective COX-2 inhibitors. Diclofenac is the most commonly used NSAID in our study, which has raised the question of whether diclofenac has a predisposition to cause DD or if diclofenac is just commonly used. All of the 16 patients known to have taken sustained-released diclofenac have diaphragm formation in the colon. Because these NSAIDs have a longer half-life, they are more likely to reach the colon before they are entirely digested. All NSAIDs were taken orally except in four cases involving suppositories. These may reflect an interaction of local and systemic effects [16].

Dosage is one factor affecting the plasma concentration of NSAIDs. In general, DD has been associated with high doses taken daily [9, 27]. Interestingly, our case has no indication for taking NSAID; more than 40 tablets of compound aminopyrine phenacetin were taken by mistake while drunk one month prior to the onset. Another important factor is how long it takes for DD to develop in a patient taking NSAIDs. Most patients take NSAIDs on a long-term basis. The duration of NSAIDs use varies from 2 months to 25 years in our study, but 133 (96%) patients had taken NSAIDs for more than one year. However, two patients have taken NSAIDs for only 2 months.

Diaphragm-like strictures can occur anywhere along the whole gastrointestinal tract. However, the majority of instances are located in small bowel [14]; 1/3 have been found in the colon [16]. In our study, 59.7% of the lesions were seen in the small bowel and 30.2% in the colon. Many reports state that small bowel DD is located predominantly in the ileum [28, 29]. Our study showed that 57.9% of small bowel DD were observed in the ileum. It may be because of the differences in the bacterial flora and immune system between the jejunum and ileum [5]. The terminal ileum is frequently spared [16]. In our study, terminal ileum is involved in only five cases. Colonic DD usually involves the right colon [30–33]. Our study also demonstrates that over 90% of colonic DD occur in the right colon and are mainly located in the ascending colon. The diaphragm in the rectosigmoid junction is the most distal lesion reported to date.

The diagnosis of DD is frequently made after an extensive workup that includes gastrointestinal radiology, endoscopy, and laparotomy. Blood tests may reveal anemia and hypoalbuminemia. For the diagnosis of DD, conventional gastrointestinal radiological techniques are inaccurate. Plain abdominal X-ray is usually unhelpful. Barium studies may show the diaphragms [27, 34, 35], but they are as easily overlooked as the thin-walled diaphragms resembling exaggerated plicae circulares [36, 37]. CT scanning may show a degree of obstruction but is unable to identify the thin diaphragms.

The upper gastrointestinal tract and the large bowel can be evaluated by EGD and colonoscopy. In our study, the majority of gastric, duodenal, and colonic DD were diagnosed by EGD and colonoscopy. The advent of CE and DBE may facilitate evaluation of the small bowel. The first diagnosis of NSAID-induced small intestinal DD through CE was reported by Yousfi et al. [38]. CE is diagnostically effective but has a significant risk of capsule retention and precipitating bowel obstruction [9, 15, 27]. Under the circumstances, laparotomy or DBE usually would be required to remove the retained capsule, so it should be used cautiously. In all 36 patients who underwent CE, retained capsule was retrieved by laparotomy in 31 (86%) patients and by DBE in 4 patients. DBE is a valuable and minimally invasive technique for the detection of diaphragm-like stricture, and endoscopic treatment is possible [39–41]. But it is technically difficult, demanding, time consuming, expensive, and not widely available and provides poor anatomical localization of diseased segments [20]. Preoperative diagnosis of small bowel DD is relatively difficult because most conventional gastrointestinal radiological techniques are unable to discern the diaphragms and limitations of endoscopy. In our study, small bowel DD were diagnosed by CE and/or DBE in 39 (41.5%) patients; 52.1% of small bowel DD were diagnosed at laparotomy. During laparotomy, the surgeon has the added advantage that the small bowel can be palpated, and a diaphragm may be felt slightly thickened. But even so, the lesion may be missed as it affects only the mucosa and submucosa leaving an intact muscularis propria and serosa [16, 28, 42], so that meticulous palpation is essential to make the diagnosis. Furthermore, intraoperative enteroscopy has been used to explore and assess the extent of the lesion [17, 28, 29, 38, 43]. Compared with laparotomy, the role of laparoscopy appears to be limited because a diaphragm may be apparent only by slight decrease in extraluminal diameter and serosal discoloration [15], as the bowel may look deceptively normal. In our study, only 3 cases were diagnosed by laparoscopy.

DD is intraluminal characterized by the presence of multiple (occasionally single [43]), thin, concentric, circumferential, and diaphragm-like mucosal projections narrowing the intestinal lumen from an approximately normal diameter to a pinhole causing varying degrees of obstruction and dividing the bowel lumen into a series of short compartments. This may make the bowel manifest as segmentation in gross specimens and resemble a string of sausages [5, 20]. The diaphragm-like strictures were often accompanied with varying degrees of erosion or ulceration [10, 19, 27], as this accounts for the chronic blood loss observed. The histopathologic characteristics of the DD are submucosal fibrosis as observed in our case [44]. Unlike Crohn's disease, DD does not affect the full thickness of the bowel wall. The muscularis propria, serosa, and mesentery usually are spared and the adjacent muscularis mucosae are interrupted and partially incorporated into the fibrotic process [16, 45]. Granulomata, which is the histopathologic characteristics of Crohn's disease, is not identified in DD [46].

The management of DD in most reported cases is segmental resection of the involved intestine, especially for the patients with DD in the small bowel. Intestinal resection was formerly the only option. Moreover, most patients underwent resection because multiple lesions were located close together along the intestine [15]. Recently, with the development of endoscopic techniques (such as DBE), endoscopic balloon dilation could be considered as an alternative option for DD [47, 48]. Given the histological feature of the DD, the risk of intestinal perforation with endoscopic balloon dilation would be low. In our study, endoscopic balloon dilation was performed in 22 patients. As with any NSAID-induced disease, the discontinuation of the NSAIDs is essential for the treatment of DD. In 18 patients for whom discontinuation of NSAIDs was the only treatment, discontinuation was associated with an improvement in symptoms. The prognosis should be good if the NSAIDs can safely be withdrawn. However, long-term cessation of NSAIDs is frequently impossible for patients with chronic arthritis or requiring antiplatelet therapy [5]. The benefit of continuing with NSAIDs may outweigh the risk of GI injury for some patients, as this needs to be considered on a case-by-case basis [46]. The use of prostaglandin derivatives (such as misoprostol) may protect against NSAID-induced GI damage, so their concomitant use should be considered in patients who are particularly at risk of NSAIDs associated GI complications, but NSAIDs are unable to be withdrawn [49]. In our study, one patient took misoprostol and ornoprostil, respectively. Recurrence of symptoms following surgical resection may occur in up to 50% of patients. This is due to either the surgeon's failure to appreciate the extent of the lesions at the initial operation or a true recurrence due to continued use of NSAIDs [10]. In our study, two patients had a relapsing course, because they resumed or continued to take NSAIDs following surgery. So, follow-up is important for timely identification and treatment of the recurrent diaphragms.

## 5. Conclusion

DD is a rare but increasingly recognized complication of NSAID usage. The pathogenesis is certainly multifactorial, but still not entirely clear. It can result in GI bleeding and obstruction. Diagnosis of DD often requires endoscopy, gastrointestinal radiological techniques, and even laparotomy. Management mainly includes discontinuation of NSAIDs, surgical resection, and endoscopic balloon dilatation. Appropriate treatment will vary with each individual. With the prevalence of NSAID usage, clinicians may encounter it more frequently. It should be considered in the differential diagnosis of patients with NSAIDs medication history and present with GI obstruction of unclear etiology as to timely diagnosis and treatment. In the future, further studies are needed to elucidate the incidence and pathogenesis of NSAID-induced DD, the related risk factors, the development of improved diagnostic techniques and treatment, and the possibility of effective medication.

## Figures and Tables

**Table 1:** Clinical manifestations in patients with NSAIDs-induced DD.

Clinical manifestations	(*n* = 155)
GI obstruction	113 (72.9%)
Abdominal pain	64 (56.6%)
Nausea and/or vomiting	40 (35.4%)
Abdominal distension	20 (17.7%)
GI bleeding	102 (65.8%)
Occult bleeding	73 (71.6%)
Overt bleeding	22 (21.6%)
OGIB	16 (15.7%)
Other	
Weight loss	28 (18.1%)
Diarrhea	22 (14.2%)
Hypoalbuminemia	21 (13.5%)
Constipation	16 (10.3%)
Acute onset of perforation	4 (2.6%)

**Table 2:** Type of NSAIDs used in patients with NSAIDs-induced DD.

NSAIDs	(*n* = 144)
Traditional NSAIDs	121 (84.0%)
Diclofenac	47 (32.6%)
Aspirin	36 (25.0%)
Indomethacin	22 (15.3%)
Ibuprofen	20 (13.9%)
Naproxen	11 (7.6%)
Paracetamol	7 (4.9%)
Azapropazone	5 (3.5%)
Phenylbutazone	4 (2.8%)
Loxoprofen	4 (2.8%)
Sulindac	3 (2.1%)
Selective COX-2 inhibitor	38 (26.4%)
Piroxicam	16 (11.1%)
Rofecoxib	7 (4.9%)
Meloxicam	5 (3.5%)
Celecoxib	4 (2.8%)
Etodolac	4 (2.8%)
Nabumetone	2 (1.4%)
Tenoxicam	1 (0.7%)
Nimesulide	1 (0.7%)
Other	
Compound aminopyrine phenacetin	1 (0.7%)

**Table 3:** Examination methods.

Examination methods	(*n* = 158)
Endoscopy	
EGD	69 (43.7%)
Colonoscopy	72 (45.6%)
Sigmoidoscopy	9 (5.7%)
CE	36 (22.8%)
Enteroscopy	18 (11.4%)
Gastrointestinal radiology	
Barium study	
Upper gastrointestinal tract series	17 (10.8%)
Small bowel follow-through/small bowel enema	59 (37.3%)
Barium enema	29 (18.4%)
CT	39 (24.7%)
Plain abdominal X-ray	23 (14.6%)
Abdominal angiography	10 (6.3%)
Nuclear tagged red blood cell scan	5 (3.2%)
Laparotomy	62 (39.2%)
Intraoperative enteroscopy	7 (4.4%)
Diagnostic laparoscopy	5 (3.2%)
